# Editorial: Biology of lysosome-related organelles

**DOI:** 10.3389/fcell.2026.1808179

**Published:** 2026-02-24

**Authors:** Cédric Delevoye, Duarte C. Barral, Subba Rao Gangi Setty

**Affiliations:** 1 Université Paris Cité, INSERM UMR-S1151, CNRS UMR-S8253, Institut Necker Enfants Malades, Paris, France; 2 iNOVA4Health, NOVA Medical School, Faculdade de Ciências Médicas, NMS, FCM, Universidade NOVA de Lisboa, Lisboa, Portugal; 3 Department of Microbiology and Cell Biology, Indian Institute of Science, Bangalore, India

**Keywords:** epidermal lamellar body, lysosome-related organelle, melanosome, platelet α-granule, secretory granule, Weibel-Palade body and secretory granule

## Introduction

Lysosome-related organelles (LROs) originate from the endo-lysosomal system with the contribution from the post-Golgi secretory pathway. While LROs share features with lysosomes, they contain unique cargo, morphologies and functions to support cell-type specific functions. Around 15 *bona fide* LROs have been described in vertebrates, with skin melanosomes serving as a classical model. When their biogenesis, secretion, or function is disrupted, a range of genetic diseases can arise, underscoring their importance in physiology (Bowman et al., 2019; Delevoye et al., 2019).

This Research Topic on the “Biology of LROs” brings together diverse members of the LRO group, including melanosomes (from both skin epidermis and retinal pigment epithelium–RPE), epidermal lamellar bodies (LBs), platelets α-granules, Weibel-Palade bodies (WPBs) from vascular endothelial cells, and secretory granules (SGs) from mast cells. Beyond LROs, the Research Topic broadens the view on endolysosomal-related processes, like autophagy and late endosome/lysosome dynamics, which help defining the cellular context of LRO specialization. In this editorial, we introduce the contributions that highlight the current understanding of LRO biology, from biogenesis to human genetic diseases associated with their dysfunction.

The perspective by Barral et al. reviews current insights into melanosome biogenesis. Melanocytes produce melanosomes, which synthesize and store melanin pigments before being transferred to keratinocytes, where they form supranuclear caps that shield genomic DNA from UV radiation-induced damage. Melanosome biogenesis follows a characterized sequential maturation process, and defects in this pathway underlie multiple hypopigmentation disorders, although therapeutic targeting remains elusive. Interestingly, melanosomes in epidermal melanocytes or RPE exhibit key differences. Unlike its skin counterparts, RPE melanosomes form early in the development, persist for life, and combine pigmentary role with lysosome-like degradative features. Finally, beyond skin and eye, the diversity of melanin types and melanocyte lineages underscores broader roles in cardiac, auditory and visual physiology.

The mini-review by Doncheva et al. further details distinctive features of RPE melanosomes compared with those of melanocytes. Although synthesized embryonically, RPE melanosomes follow biogenesis and trafficking pathways similar to skin melanosomes. Defects in genes regulating RPE melanosome positioning underlie genetic disorders, while age-related melanosome dysfunction contributes to retinal degenerative diseases such as age-related macular degeneration (AMD). RPE melanosomes form membrane contacts with mitochondria and/or the endoplasmic reticulum, enabling Ca^2+^ exchange and reactive oxygen species quenching, and thus cellular homeostasis. In parallel, RPE lysosomes facilitate the digestion of photoreceptor outer segments, with failure in this pathway resulting in the accumulation of the toxic ageing pigment lipofuscin. Fusion of lipofuscin-containing lysosomes with melanosomes form melanolipofuscin, promoting melanin-dependent lipofuscin degradation through chemiexcitation. Beyond these roles, RPE melanosomes also exert protective effects in AMD, highlighting how long-lived RPE melanosomes link cell-type specific functions to retinal diseases.

Focusing on platelets, the mini-review by Ambrosio and Di Pietro discusses the biogenesis of α-granules, an LRO derived from megakaryocytes (MKs). These granules are central to hemostasis and also store factors involved in angiogenesis and inflammation. As with other LROs, α-granule formation relies on sorting and recycling endosomes, which act as membrane transport intermediates, a process well illustrated using induced pluripotent stem cell -derived MKs. This cellular system has become a powerful model for dissecting how ubiquitous membrane trafficking machinery is adapted for α-granule biogenesis. Such specialization could rely on the MK-enriched protein NBEAL2, which emerges as a key adaptor coordinating endosomal membrane dynamics and cargo sorting. Together, mutations in several of these components cause human bleeding disorders.

The review by Leprince and Simon focuses on epidermal LBs, the LROs in keratinocytes of the *stratum granulosum* that are essential for skin barrier function. LBs store lipids, proteases, and antimicrobial peptides, which together maintain the hydrophobic properties, structural integrity, and defense capacity of the uppermost skin layer, the *stratum corneum*. LB dysfunction leads to barrier defects and an ichthyotic phenotype marked by dry, thickened, and scaly skin. Despite their importance, the molecular mechanisms regulating LB biogenesis and trafficking remain poorly characterized. As for other LROs, endosomal components such as Rab11a, Myosin Vb and the CHEVI tethering complex have been implicated in LB trafficking. Further work is therefore needed to clarify LB biology and the etiology of diseases involving their dysfunction, including atopic dermatitis and active plaque psoriasis.

The perspective by Mahanty further highlights the scarce knowledge on the biogenesis mechanisms of LBs, emphasizing their origin at the *trans*-Golgi network (TGN), and discusses the models for LB structure/morphology and secretion mechanisms. Furthermore, it pinpoints candidate regulators of LB biogenesis and trafficking based on evidence from disease models. Finally, it discusses the limitations of current models used to study LB biology and underscores the need to develop advanced models, such as 3D skin organoids that can be manipulated and accurately reflect the differentiation state of *stratum granulosum* keratinocytes.

The mini-review by Terglane and Gerke addresses the biogenesis, transport, and regulated secretion of WPBs, the specialized LROs of vascular endothelial cells. Unlike most LROs, WPBs originate at the TGN, and their biogenesis is largely dictated by post-translational maturation of the prothrombotic and hemostatic glycoprotein von-Willebrand factor (VWF). During this process, VWF undergoes complex glycosylation, polymerization and compaction, shaping WPBs into their characteristic cigar-like morphology. WPBs move along microtubules and can be exocytosed upon endothelial cell activation during inflammation, infection, or vascular injury, therefore modifying the endothelial surface to promote leukocyte and platelet adhesion. The authors detail key regulatory steps, including BLOC-2-dependent V-ATPase transport to WPBs, Rab27a-Slp2a-PI(4,5)P_2_-mediated WPB tethering to the cell surface, and actomyosin-driven control of VWF release. Despite these advances, important questions remain, notably how WPB cargo release can be precisely modulated using pharmacological agents.

The review article by Montero-Hernandez et al. expands the LRO landscape by exploring secretory lysosomes, also called SGs, in mast cells of the hematopoietic lineage. These organelles store inflammatory mediators such as histamine, serotonin, and lysosomal enzymes, which are released through regulated exocytosis during immune responses. SG biogenesis depends on close coordination between the endocytic and exocytic pathways at the interface of the TGN and early/recycling endosomes. As they mature, SGs undergo bidirectional transport along microtubules before SNARE-dependent secretion. Understanding SG biology provides key insights into how cells translate external physiological stimuli, or ‘danger signals’, such as toxins and microbes, into rapid, regulated degranulation and inflammatory responses, opening avenues for therapeutic intervention in allergic manifestations.

In continuation, the review by Sagi-Eisenberg presents the current view on SGs produced by mast cells. Interestingly, a cohort of SGs exhibits more of the typical lysosome features. SGs originate from the TGN, grow in size by homotypic fusion, and acquire components by fusing with endosomes or amphisomes. Thus, they differ in their content and morphology and are classified into Type I, II and III based on ultrastructural studies. Mast cells are mostly present at the interface between the external environment and the internal milieu. Upon activation (during allergy and inflammation) of mast cells, the stored inflammatory mediators of SGs such as histamine, proteoglycans and proteases, are released through exocytosis. SGs are enlarged or abnormal in diseases such as Chediak-Higashi and Hermansky-Pudlak syndromes, respectively. The role of serglycin and acidic pH in maintaining SG function, as well as the changes that occur during ageing, are also highlighted.

The opinion article by Wang et al. examines how autophagy regulates the homeostasis of LROs. The authors discuss the role of selective autophagy of melanosomes–melanophagy–in the degradation of this type of LRO. Moreover, they highlight the role of non-canonical autophagy in the processing of antigens in major histocompatibility complex class II compartments, as well as in the secretion of WPBs from endothelial cells, LBs from type II alveolar epithelial cells, and SGs from mast cells. Several unanswered questions remain, including whether selective autophagy exists for LROs other than melanosomes, the LRO cargoes and autophagy receptors at play for each type of LRO, and how canonical and non-canonical autophagy are coordinated within the same LRO-producing cell.

Finally, the review by Bakker et al. discusses how late endosomes/lysosomes and LROs are transported intracellularly to reach their correct destinations and perform their functions. Although these organelles share common features, they also display specific transport requirements. Their movement depends on coordinated interactions with molecular motors: dynein and kinesin drive long-range, bidirectional transport along microtubules, while myosins enable short-range motion along actin filaments. This coupling to the cytoskeleton is finely regulated by multiple systems, including the tubulin code, microtubule-associated proteins, as well as Rab GTPases and their effectors. Proper coordination is essential, as defects in these transport pathways are linked to skin, neurological, and immune disorders.

## Conclusion

This Research Topic on the “Biology of LROs” highlights the remarkable diversity of LROs and the shared principles that underlie their formation, maturation, trafficking, and secretion in specialized cell types. Together, the contributions illustrate how variations on endo-lysosomal and secretory pathways lead to the formation of LROs with distinct structures, functions and contents, and how their dysregulation contributes to various human disorders. While not exhaustive, this Research Topic reflects the breadth and growing complexity of LRO biology, from fundamental cell biology to physiological and pathological aspects. We hope this Research Topic will stimulate further exploration into how LRO identity is established, maintained, and altered in disease and ageing, ultimately opening new avenues for therapeutic intervention.
